# Amblypygids: Model Organisms for the Study of Arthropod Navigation Mechanisms in Complex Environments?

**DOI:** 10.3389/fnbeh.2016.00047

**Published:** 2016-03-08

**Authors:** Daniel D. Wiegmann, Eileen A. Hebets, Wulfila Gronenberg, Jacob M. Graving, Verner P. Bingman

**Affiliations:** ^1^Department of Biological Sciences, Bowling Green State UniversityBowling Green, OH, USA; ^2^J.P. Scott Center for Neuroscience, Mind and Behavior, Bowling Green State UniversityBowling Green, OH, USA; ^3^School of Biological Sciences, University of NebraskaLincoln, NE, USA; ^4^Department of Neuroscience, University of ArizonaTucson, AZ, USA; ^5^Department of Psychology, Bowling Green State UniversityBowling Green, OH, USA

**Keywords:** amblypygid, mushroom bodies, multimodal sensory integration, navigation mechanisms, *Phrynus*

## Abstract

Navigation is an ideal behavioral model for the study of sensory system integration and the neural substrates associated with complex behavior. For this broader purpose, however, it may be profitable to develop new model systems that are both tractable and sufficiently complex to ensure that information derived from a single sensory modality and path integration are inadequate to locate a goal. Here, we discuss some recent discoveries related to navigation by amblypygids, nocturnal arachnids that inhabit the tropics and sub-tropics. Nocturnal displacement experiments under the cover of a tropical rainforest reveal that these animals possess navigational abilities that are reminiscent, albeit on a smaller spatial scale, of true-navigating vertebrates. Specialized legs, called *antenniform legs*, which possess hundreds of olfactory and tactile sensory hairs, and vision appear to be involved. These animals also have enormous mushroom bodies, higher-order brain regions that, in insects, integrate contextual cues and may be involved in spatial memory. In amblypygids, the complexity of a nocturnal rainforest may impose navigational challenges that favor the integration of information derived from multimodal cues. Moreover, the movement of these animals is easily studied in the laboratory and putative neural integration sites of sensory information can be manipulated. Thus, amblypygids could serve as model organisms for the discovery of neural substrates associated with a unique and potentially sophisticated navigational capability. The diversity of habitats in which amblypygids are found also offers an opportunity for comparative studies of sensory integration and ecological selection pressures on navigation mechanisms.

## Introduction

The neural substrates that underlie complex behavior remain poorly understood in any animal. The elucidation of behavioral and neural mechanisms by which animals navigate, in particular, has been identified as one of the most important scientific challenges of our time (Kennedy and Norman, 2005). Indeed, navigation could be exploited for the study of sensory system integration and its relation to complex behavior, a core, unresolved issue in neuroscience and systems biology (Wiener et al., 2011). For such a goal to be realized, however, it will be necessary to avoid temptations to reduce navigational control to a handful of sensory cues, studied as independent information channels. New model systems may also be warranted, systems that are both tractable and sufficiently complex to thwart the utility of path integration and devalue information derived from a single sensory modality. Ideally, any new model system should also include taxonomically related species that inhabit distinct environments so that comparative approaches can be used to identify ecological selection pressures associated with the neural architecture of navigation behavior.

Navigation behavior has been studied intensively in a variety of terrestrial arthropods, like fiddler crabs, dung beetles, spiders and notably, desert ants (reviewed by Dyer, 1998; Cheng, 2012; Perry et al., 2013; Ortega-Escobar and Ruiz, 2014). The neuroethology of visually guided behavior in the Saharan ant *Cataglyphis* is especially well studied and *Cataglyphis* has become the standard model for the study of arthropod navigation (Wehner, 1984, 2003). These focal animals inhabit largely two-dimensional environments and predictably, their navigation strategies share a number of properties. For instance, fiddler crabs, wolf spiders and desert ants use *path integration* to relocate a shelter, where an accumulator encodes the position of the navigator relative to a goal from continually updated directional and distance information (Layne et al., 2003a,b; Wehner, 2003; reviewed by Collett and Collett, 2000). Direction is frequently determined, as in *Cataglyphis*, with respect to a time-compensated sun compass and the distance an individual travels in a particular direction is computed from idiothetic, proprioceptive cues (Mittelstaedt and Mittelstaedt, 1982; Wehner, 2003; Reyes-Alcubilla et al., 2009). Thus, a desert ant or a fiddler crab can traverse a circuitous route in search of food in an unfamiliar landscape and return to its nest or burrow on a straight-line trajectory. In fact, *Cataglyphis fortis* follows a path-integrated return route that is approximately its full outbound distance and then transitions to a systematic search for its nest (Merkle et al., 2006).

In habitats that are largely two-dimensional and bereft of distinctive landmarks, like the salt pans of Tunisia, path integration is an effective and probably essential navigation strategy. But the habitats and activity patterns of many arthropods limit the utility of strategies that rely chiefly on an idiothetic distance accumulator or visual cues. The semi-arid habitat of the Central Australian desert ant *Melophorus bagoti*, for instance, is moderately cluttered, with scattered tussock plants, and *M. bagoti* primarily follows stereotypical, learned routes (Kohler and Wehner, 2005). Indeed, *M. bagoti* path integrates less than half its outbound distance in unfamiliar areas and then transitions to search for visual cues associated with familiar routes (reviewed by Cheng et al., 2009). The floor of a rainforest is even more unlike the smooth sands of deserts or beaches and inhabitants of rainforests have evolved alternative solutions to navigation problems. Many ants, for example, use pheromones and manicured trails as navigational guides (Jackson et al., 2004; Collett and Collett, 2007). Nomadic army ants, which use a mobile home, avoid the problem of navigation back to a nest altogether (Couzin and Franks, 2002). Nocturnal animals are further challenged by light levels many orders of magnitude lower than those experienced by diurnal animals, which limit spatial resolution and may constrain activity (Kelber et al., 2006; Somanathan et al., 2008). Many insects have, however, evolved specialized compound eyes and nervous systems that allow them to use polarized moonlight—a million times dimmer than polarized sunlight—as a compass cue or to memorize canopy patterns that guide routes to and from their nests (Warrant and Dacke, 2010; el Jundi et al., 2015).

In complex environments cue reliability probably also poses problems for navigators. For instance, a celestial cue could be highly visible in some locations of a forest and at other locations, be obscured entirely by the canopy. Thus, in structurally complex habitats, it seems, navigation solutions that rely on multiple sensory modalities might be especially advantageous or, perhaps, even necessary. Interestingly, *C. fortis*, a species once thought to rely solely on vision and path integration to find its nest, can be trained to pinpoint a nest entrance with an odor cue and is better able to locate the nest entrance when trained with an odor and visual landmarks than when trained on either cue separately (Steck et al., 2009, 2011).

The enhanced navigational performance by *C*. *fortis* when visual and olfactory cues are available implies that these cues are, at some level, *integrated*. In arthropods, the integration of multimodal information likely occurs in the mushroom bodies, brain centers found in the first brain segment of all arthropods and their common ancestors (Kenyon, 1896; Strausfeld et al., 2006; Brown and Wolff, 2012; Strausfeld, 2012; Wolff et al., 2012). In insects, the mushroom bodies are further implicated in behavioral plasticity and olfactory learning and memory (reviewed by Heisenberg, 2003; Strausfeld, 2012). In a study of cockroaches, the mushroom bodies were also implicated in *spatial memory* (Mizunami et al., 1998). Indeed, the relative size of mushroom bodies in arthropods is argued to correlate positively with ancestral navigational demands (Jacobs, 2012). However, recent studies of insects—notably, *Drosophila*—implicate the *central complex* as a center for spatial orientation and memory (Liu et al., 2006; Neuser et al., 2008; reviewed by Pfeiffer and Homberg, 2014). In arachnids, little is known about the function of the mushroom bodies or the *arcuate body*, a chelicerate neuropil that likely shares a common evolutionary origin with the central complex, although the latter brain region appears to integrate visual information (Loesel et al., 2011; Menda et al., 2014; reviewed by Strausfeld, 2012).

Here, we discuss some recent research on navigation by amblypygids, nocturnal arachnids that could prove to be invaluable organisms for studies of the neurobiological foundations of complex navigation behavior in arthropods. Indeed, amblypygids exhibit navigation abilities that are seemingly comparable to those of some vertebrates and, incidentally, have mushroom bodies that are larger, relative to their body size, than any other studied arthropod (Strausfeld et al., 1998).

## Ecology and Sensory Biology of Amblypygids

Amblypygids are an arachnid order comprised of about 160 species, distributed worldwide in the tropics and subtropics (reviewed by Weygoldt, 2000; Harvey, 2007; Chapin and Hebets, in press). The majority of species are found in rainforests, but many species are cave dwellers—troglophiles—and a few are even found in savannahs or deserts. The rainforest species are strictly nocturnal and in the day hide in hollow trees, in burrows of small mammals or in rock crevices.

**Figure 1:**
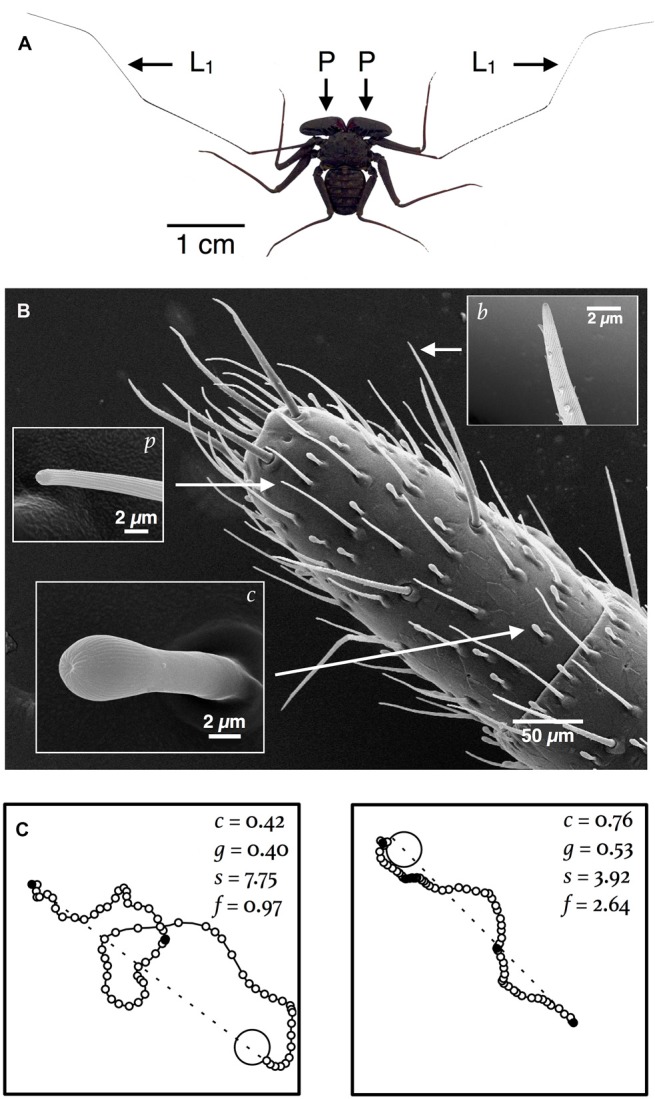
**Navigation by amblypygids is hypothesized to rely on sensory information derived from sensilla on the antenniform legs. (A)** Image of *P. marginemaculatus* that shows the (L_1_) antenniform legs and (P) raptorial pedipalps. **(B)** SEM of the distal tip of an antenniform leg of *P. marginemaculatus*, which shows three types of sensilla: *c*, club sensillum (contact chemosensory); *p*, multiporous sensillum (olfactory); and *b*, bristle (mechanosensory and contact chemosensory). **(C)** In the laboratory subjects readily utilize an artificial shelter. Shown here are nocturnal return routes for two subjects (recorded every 2 s) in a 1-m^2^ arena. Four kinematic variables used to characterize the paths are listed: *c*, a circuity index, is the straight line distance (dashed line) from the start point of the return route to the shelter (large open circle) divided by the actual distance traveled; *g*, a goal orientation index, measures the directedness of the path with respect to the shelter (described in Bak-Coleman et al., 2013); *s*, the mean linear speed (mm s^-1^); and *f*, the frequency of pauses (per minute) in motion (small filled circles) on the route to the shelter.

Amblypygids are flat and spider-like, with large raptorial pedipalps that are used to capture prey, fight with rivals and court prospective mates (Figure 1A). Unlike true spiders, amblypgids walk on six legs. Their anterior pair of legs, called *antenniform legs*, are elongated sensory structures—especially so in troglophile species—that may span more than fifteen times the length of their body (Igelmund, 1987; Weygoldt, 2000). The antenniform legs are highly articulated and covered with hundreds of mechanosensory, chemosensory and possibly, humidity-sensing sensilla (Figure 1B; Foelix, 1975; Beck et al., 1977; Santer and Hebets, 2011). Amblypygids can readily differentiate between fine-scale textures and, presumably by contact chemoreception, discriminate kin from non-kin (Walsh and Rayor, 2008; Santer and Hebets, 2009). Furthermore, multiporous sensilla, located on the distal tips of the antenniform legs, have a confirmed olfactory function, which implies that important aspects of amblypygid behavior may, unlike the behavior of most true spiders, be guided by olfaction (Figure 1B; Hebets and Chapman, 2000). Experiments that we recently conducted with *Phrynus pseudoparvulus*, discussed momentarily, suggest that olfaction may be important for navigation at night by amblypygids that inhabit rainforests (Hebets et al., 2014a,b).

Amblypygids typically possess eight small, single-lens eyes: two medial eyes located near the anterior carapace margin, which are diminutive or absent in some troglophile species, and two groups of three lateral eyes positioned near the anterolateral carapace margins (Weygoldt, 2000). In *Phrynus* *marginemaculatus*, the two medial eyes have microvilli that are oriented in a manner that suggests to us that these eyes are, like the specialized receptors of the *dorsal rim* areas in many insect eyes, sensitive to skylight polarization (Gebhardt, 1983). Furthermore, the medial eyes of *P*. *marginemaculatus* are capable of rudimentary image formation and in addition to photoreceptors with peak sensitivity near 500 nm, may have dedicated UV photoreceptors (Graving et al., in preparation). Little else is known about the physiology or optics of amblypygid eyes, apart from an unpublished thesis by Gebhardt (1983; but see Paulus, 1979).

## Navigation Behavior of Amblypygids in the Field

Amblypygids that inhabit rainforests emerge from their refuges—typically crevices of tree buttresses—at night to hunt for invertebrate prey. They are sit-and-wait predators and can be relocated near the base of the same tree night after night for a period of weeks or months (Beck and Görke, 1974; Weygoldt, 1977). But individuals occasionally wander distances of 30 m or more over a period a several nights and later return to the tree on which they were originally sighted (Hebets, 2002).

Beck and Görke (1974) were the first researchers to document amblypygid navigation behavior. They displaced nine *Heterophrynus batesii*, a large Amazonian species, distances of 2.5–7.5 m and one subject 10 m when they emerged at night from tree crevices and placed them on the ground. Each individual that was displaced 7.5 m or less returned to the tree on which it was captured on the same night that it was displaced. The subject that was displaced 10 m returned sometime between 2 to 5 nights after it was displaced. Beck and Görke (1974) secondarily displaced one subject 3.5 m—to its original release site—after the distal 30–50 articles of its antenniform-leg tarsi had been clipped. They searched for several nights afterward at the tree from which it was displaced, but it never returned.

These simple experiments yielded an important result: path integration, a seemingly ubiquitous navigation strategy in terrestrial arthropods that inhabit largely two-dimensional environments, is *not* necessary for successful navigation by adult *H. batesii*. The performance of the animal with the distal tips of its antenniform legs clipped—the *exclusive* location of olfactory sensilla—also hints at the possibility that odors or other cues detected by sensilla on the antenniform legs are somehow involved (Weygoldt, 2000).

Nocturnal displacement experiments that we recently conducted with *P. pseudoparvulus*, a species that inhabits rainforests of Central America, provide more detailed insights into amblypygid navigation behavior. Displaced *P. pseudoparvulus*, equipped with radio transmitters, generally return in a single night to the tree from which they are captured if they are displaced a distance less than 10 m, like *H. batesii* (Hebets et al., 2014b). Longer displacements typically involve a temporary residency at another tree (or in a burrow) and individuals are capable of successful navigation from displacement distances as far as 25 m. Experiments in which we introduced individuals to trees on which residents were removed also showed that certain trees do not simply act as attractor beacons.

Recent sensory deprivation experiments also suggest that inputs from the antenniform legs and, perhaps, vision contribute to successful navigation by *P. pseudoparvulus* (Hebets et al., 2014a). In particular, individuals almost never return to the tree from which they are captured after an 8-m displacement if the sensory sensilla on the distal tips of the antenniform legs are made non-functional. The return rate for vision deprived individuals after a displacement of 8 m, in contrast, appears to be only moderately impaired.

## Behavior in the Laboratory

The study of navigation behavior at night in complex environments like a rainforest has two notable pitfalls: the environment itself hampers the manipulation of *cues* that might be involved and it limits the detail with which individuals can be tracked. Fortunately, amblypygids are also amenable to laboratory experiments, where cues can be manipulated and computer vision software can be used to quantify nocturnal movements in considerable detail.

In the laboratory, *P. marginemaculatus*, *P. pseudoparvulus* and *Paraphrynus mexicanus* (which regularly inhabits caves) all readily utilize an artificial shelter and wander nightly in an arena. Figure 1C shows the inbound paths—return routes to an artificial shelter—for two *P. marginemaculatus* subjects, with values for various kinematic variables used to quantitatively characterize the paths. The detail with which movements can be measured in the laboratory should allow researchers to detect even subtle changes in behavior caused by cue manipulations (Drai et al., 2000; Wallace et al., 2006; Benjamini et al., 2010; Donelson et al., 2012; Dell et al., 2014).

## Integration of Navigational Sensory Information

In comparison to insects, relatively little is known about how sensory information is processed in arachnids (Strausfeld, 2012). What is known is based largely on studies of spiders (e.g., Babu and Barth, 1984; Babu, 1985; Babu et al., 1985; Strausfeld and Barth, 1993). Few studies describe the central nervous system (CNS) of amblypygids in any detail beyond its coarse anatomical composition (Babu, 1965; Babu et al., 1985).

**Figure 2:**
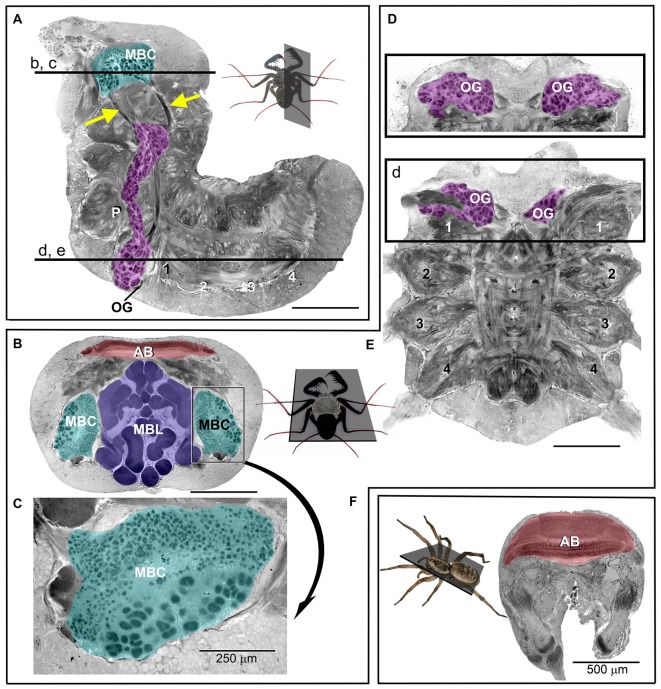
**Photomicrographs of sections through the (osmium-stained) central nervous system (CNS) of *P. marginemaculatus*. (A)** Sagital and **(B–E)** horizontal sections show (color-coded) olfactory glomeruli, OG (magenta); mushroom body calyces, MBC (cyan); mushroom body lobes, MBL (purple) and the arcuate body, AB (red). **(A,D,E)** The ventral neuromeres that supply the pedipalps (P), the antenniform leg (1) and the walking legs (2–4). Insets show the respective planes of sections and the labeled horizontal lines in **(A)** indicate the dorso-ventral depths of sections in **(B–E)**. The mushroom body calyx in **(B)** is enlarged and rotated in **(C)** to show the distinct difference in glomeruli size. The cross sectional profiles (purple) in **(B)** reveal the complex and convoluted organization of the mushroom body lobes. The olfactory glomeruli in **(D)** are shown at a level 90 μm more dorsal with respect to **(E).** Arrows in **(A)** indicate tracts that are assumed to connect olfactory glomeruli with the mushroom body calyx. **(F)** Brain section of a huntsman spider (*Olios giganteus*), which is comparable in size to *P*. *marginemaculatus*. Note the considerably larger arcuate body in the spider compared to *P*.* marginemaculatus*, shown in **(B)**. Unlabeled scale bars are 500 μm.

The CNS in arachnids is typically composed of the preoral ganglia, which form the brain proper, also called the supraesophageal ganglion (Figures 2A–C), and the fused ventral ganglia, which serve the legs and the abdomen (Figures 2D,E; Babu and Barth, 1984). Besides the brain proper, the first leg neuromeres of the ventral ganglia are of particular interest because they receive input that originates from the many mechanosensory and chemosensory sensilla on the antenniform legs (Figure 1B; reviewed in Santer and Hebets, 2011).

In amblypygids, mechanosensory information is probably organized and processed in leg neuromeres in a manner similar to spiders (Babu and Barth, 1984). How mechanosensory information is processed upstream of the ventral ganglia in arachnids is yet to be elucidated. The neuromeres of the antenniform legs further give rise to a large number of glomeruli, which presumably process chemosensory information that originates from the antenniform legs (Figure 2). These glomerular regions, which are analogous to the olfactory lobes of insects and vertebrates, are large and extend from their original ganglion into the nearby pedipalpal ganglion (Strausfeld, 2012). The precise number of amblypygid olfactory glomeruli is unknown, but we estimate that there are over 500 glomeruli per side, more than in any insect that has been studied, with the exception of forager castes of two *Camponotus* ant species (Mysore et al., 2009). The number of olfactory glomeruli across animal taxa is generally related to the sophistication of the olfactory system and to the number of odors and odorants that can be detected and discriminated. The size of individual glomeruli is hypothesized to relate to sensitivity thresholds for certain odorants, as in insects that are extremely sensitive to species-specific pheromones (reviewed by Galizia and Rössler, 2010).

The mushroom bodies appear, based on a preliminary examination, to receive massive olfactory input via a prominent tract from the olfactory glomeruli in the ventral ganglia (Figure 2A). In contrast, we did not find any tracts that connect the olfactory glomeruli to the arcuate body. In spiders, the only arachnids for which this kind of anatomical information is available, the arcuate body and mushroom bodies receive visual input (Strausfeld and Barth, 1993; Strausfeld et al., 1998). There is currently no anatomical evidence for visual input into the mushroom bodies in amblypygids, but by analogy to spiders visual input is expected. Putative input from visual centers will be mapped in future experiments by retrograde and anterograde tracing (by the injection of fluorescent tracers into the mushroom body lobes and calyces and into visual neuropils, starting at the photoreceptors, respectively). Interestingly, visual and olfactory projections to the mushroom bodies occur in certain insects, most notably in advanced Hymenoptera, which are known for their navigational capabilities (Strausfeld et al., 2009; Farris and Schulmeister, 2011).

The mushroom body lobes of amblypygids are, in comparison to insects, exceptionally large and elaborately folded (Figure 2B). These multi-lobed structures comprise much of the dorsal brain volume and are, indeed, relatively larger than those of any other studied arthropod (Strausfeld et al., 1998). The mushroom body calyces appear equally extraordinary: they are uniquely subdivided into glomeruli of two different kinds (Figures 2B,C). Insect mushroom body calyces are composed of microglomeruli, synaptic complexes that are visible at the light-microscopic level. The smaller calycal glomeruli in amblypygids seem comparable to these synaptic complexes. Their larger calycal glomeruli, however, are reminiscent of olfactory glomeruli and have no known counterparts in any other arthropod.

The organization of sensory projections from the antenniform legs and their potential integration with visual information in the mushroom bodies should provide initial clues about the function of the mushroom bodies and the distinctive architecture of the calycal glomeruli with regard to the neural integration of information that controls amblypygid navigation behavior. The arcuate body in amblypygids appears to be small compared to spiders, like the huntsman *Olios giganteus*, which exhibit a comparable lifestyle (Figures 2B,F). In insects, the central body, which likely shares a common evolutionary origin with the arcuate body, appears to be involved in spatial orientation and memory. But with no current connectivity information related to the arcuate body in amblypygids we are reluctant to speculate about its contribution to their navigation behavior. The importance of sensory integration *per se* to successful navigation can be more directly assessed via targeted lesions of putative mushroom body integration sites and other brain areas, techniques that we have recently developed (see also Menda et al., 2014).

## Conclusions

Experiments that we recently conducted in the rainforest of Central America revealed that the amblypygid *P. pseudoparvulus* has a sophisticated, nocturnal navigational system that does not rely on path integration, a common navigation mechanism used by terrestrial arthropods that inhabit simpler, largely two-dimensional environments. *P. pseudoparvulus*, like all amblypygids, have specialized sensory structures—antenniform legs—that are covered with hundreds of sensory hairs with mechanosensory and chemosensory functions. Field experiments suggest that sensory inputs from the antenniform legs play a critical role in amblypygid navigation and that visual information alone is insufficient to guide displaced *P. pseudoparvulus* back to their shelters. The results of these experiments are in general accord with our central hypothesis that navigation in complex environments is supported by the integration of information derived from multimodal sensory cues. Preliminary neurobiological results reveal that, in amblypygids, neuronal integration of olfactory information likely occurs in the mushroom bodies. In future experiments we will verify whether, as suspected, the mushroom bodies also receive inputs from visual centers. The nocturnal movement of amblypygids can be characterized in detail in the laboratory and putative neural integration sites of sensory information can be manipulated. Thus, amblypygids may serve as a model system for the study of neural substrates associated with navigation behavior in complex environments. The diversity of habitats in which amblypygids are found further offers an opportunity for comparative studies, which could reveal associations between their ecology and neuronal patterns of sensory integration and the identification of selection pressures that act on navigation mechanisms.

## Author Contributions

DDW, EAH, WG and VPB: writing, data collection and funding. JMG: writing and data collection.

## Conflict of Interest Statement

The authors declare that the research was conducted in the absence of any commercial or financial relationships that could be construed as a potential conflict of interest.
